# Supplementary figures and images for: Independent insulin signaling modulators govern hot avoidance under different feeding states
Source: PLoS Biol. 2023 Oct 17;21(10):e3002332. doi: 10.1371/journal.pbio.3002332 (PMC10581474; doi:10.1371/journal.pbio.3002332)

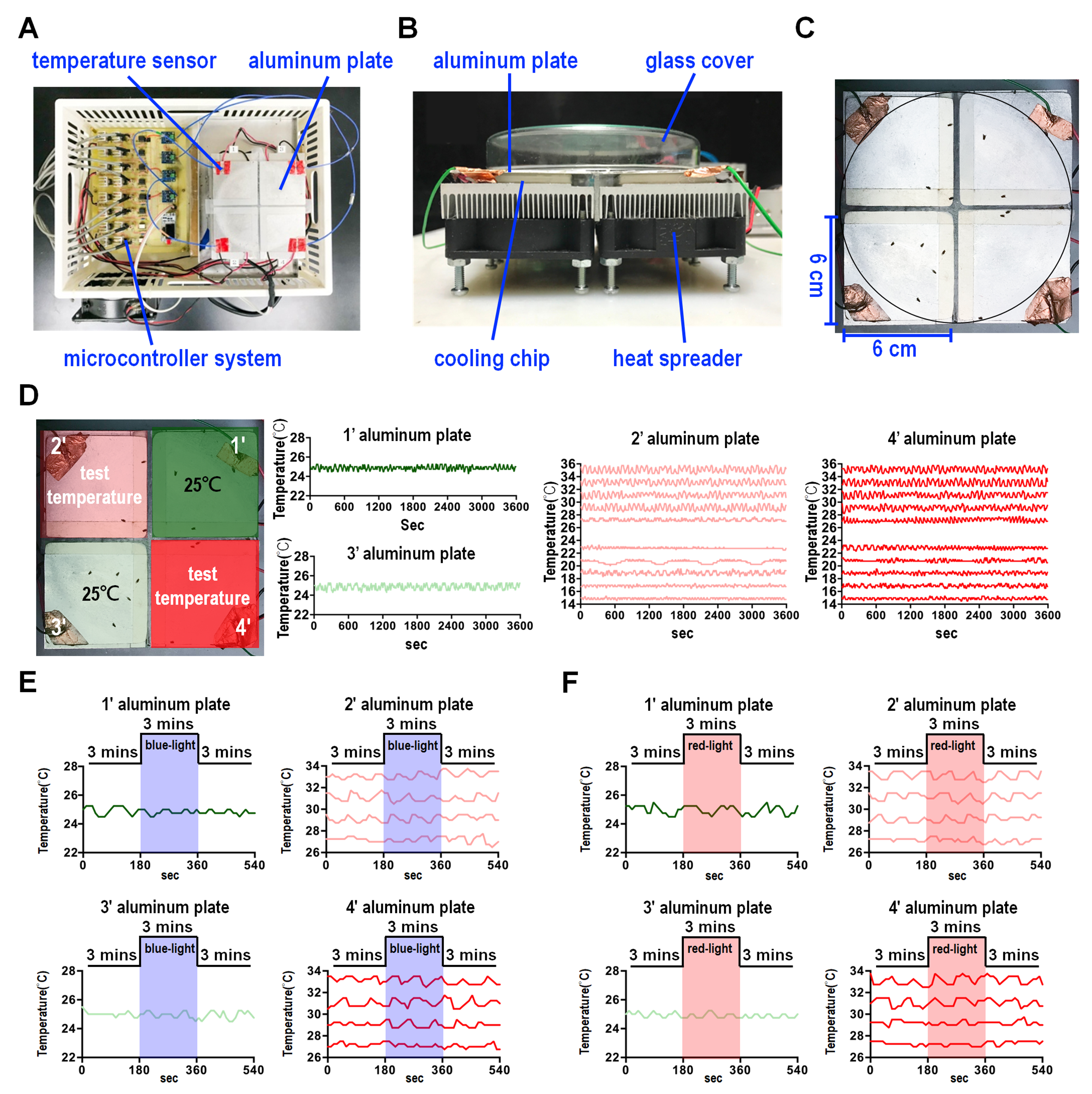

Supplement: S1 Fig — (A, B) The top view (A) and side view (B) of a thermoelectric device. The thermoelectric device contains 6 components: microcontroller system, temperature sensors, aluminum plates, cooling chips, heat spreaders, and a glass cover. The pulse-width modulation (PWM) is used to control the operating voltage of thermoelectric cooling module. The thermoelectric cooling module is used to control the temperature of aluminum plate that range from 15°C to 35°C. We set a 6 × 6 cm aluminum sheet on each thermoelectric cooling module to increase conduction velocity. For heat dissipation, we set a heat spreader with aluminum below each cooling chip. Four thermoelectric cooling modules are lined up to form an arena and temperature sensor is added in each thermoelectric cooling module. (C) The distributions of flies on the thermoelectric device during a two-choice assay experiment. (D) The accuracy and stability of the thermoelectric device has been shown. The 2’ (light-red region) and 4’ (dark-red region) quadrants were individually set to different test temperatures (15, 17, 19, 21, 23, 27, 29, 31, 33, or 35°C). The 1’ (dark-green region) and 3’ (light-green region) quadrants were set to 25°C. The temperatures on each of the 4 quadrants plates were recorded for 3,600 s. The margin of error on each aluminum plate was lower than 1°C (approximately 0.5°C) in all test temperature settings. (E, F) Three min of blue or red light irradiations did not alter the setting temperatures of the aluminum plates of the thermoelectric device. The 2’ (light-red region) and 4’ (dark-red region) quadrants were set to different test temperatures (27, 29, 31, or 33°C). The 1’ (dark-green region) and 3’ (light-green region) quadrants were set to 25°C. The temperature was recorded for 540 s on each aluminum plate, and light irradiations were added from 180 to 360 s. The margin of error on each aluminum plate was lower than 1°C in blue light (E) and red light (F) irradiation conditions. The data underlyi [file pbio.3002332.s001.tif]

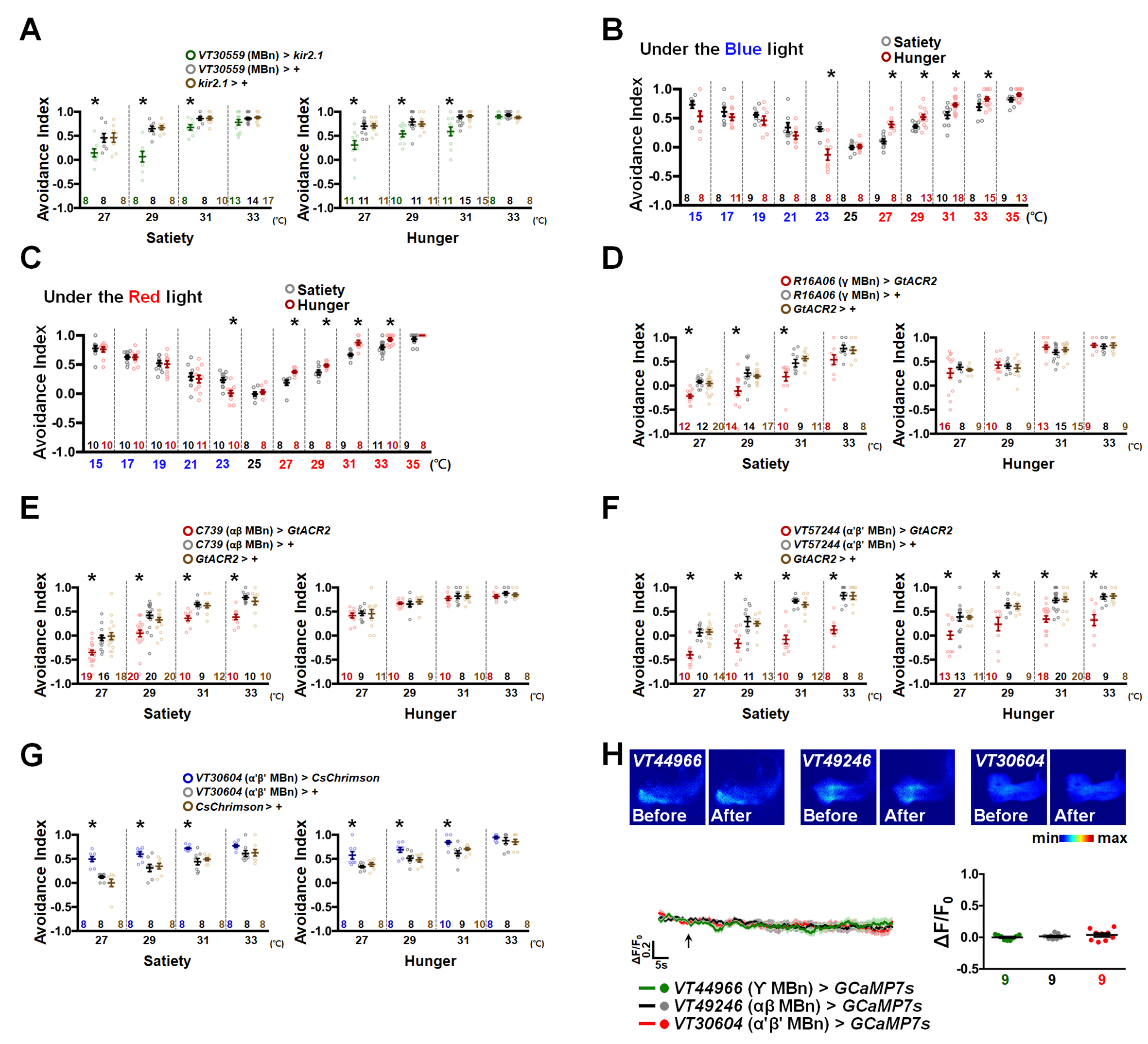

Supplement: S2 Fig — (A) Silencing MBn activity via VT30559-GAL4 > UAS-kir2.1 reduced HAB during both sated and hungry states (Satiety: P-values: 0.033, <0.0001, 0.0073, and 0.1177 from left to right; Hunger: P-values: 0.0002, 0.0097, 0.0002, and 0.4309 from left to right). (B) Hungry flies showed significantly higher HAB than sated flies under blue light irradiation (P-values: 0.0832, 0.3191, 0.2438, 0.202, 0.0006, 0.7372, 0.0003, 0.0124, 0.0058, 0.0387, and 0.0569 from left to right). (C) Hungry flies showed significantly higher HAB than sated flies under red light irradiation. (P-values: 0.7901, 0.9453, 0.8403, 0.6582, 0.0015, 0.4891, 0.002, 0.0231, 0.0004, 0.0101, and 0.0962 from left to right). (D) Silencing γ MBn activity via R16A06-GAL4 > UAS-GtACR2 reduced HAB during the sated state (Satiety: P-values: <0.0001, 0.0005, 0.0024, and 0.1123 from left to right; Hunger: P-values: 0.5773, 0.7602, 0.1986, and 0.9307 from left to right). (E) Silencing αβ MBn activity via C739-GAL4 > UAS-GtACR2 reduced HAB during the sated state (Satiety: P-values: 0.0002, 0.0001, 0.0002, and < 0.0001 from left to right; Hunger: P-values: 0.8762, 0.7292, 0.684, and 0.422 from left to right). (F) Silencing α′β′ MBn activity via VT57244-GAL4 > UAS-GtACR2 reduced HAB during sated and hungry states (Satiety: P-values: <0.0001, 0.0009, <0.0001, and <0.0001 from left to right; Hunger: P-values: 0.0009, 0.0111, <0.0001, and 0.0001 from left to right). (G) α′β′ MBn activation via VT30604-GAL4 > UAS-CsChrimson increased HAB (Satiety: P-values: <0.0001, 0.0058, 0.0005, and 0.1063 from left to right; Hunger: P-values: 0.0082, 0.0116, 0.0011, and 0.4237 from left to right). (H) Room temperature stimuli did not induce a significant calcium response in γ, αβ, and α′β′ MBn. The GCaMP intensity changes (ΔF/F0) in γ, αβ, and α′β′ MBn horizontal lobes were recorded and analyzed. There were no significant differences in GCaMP intensity in γ, αβ, and α′β′ MBn before and after room temperature stimuli (P-values: 0.9902, 0.29 [file pbio.3002332.s002.tif]

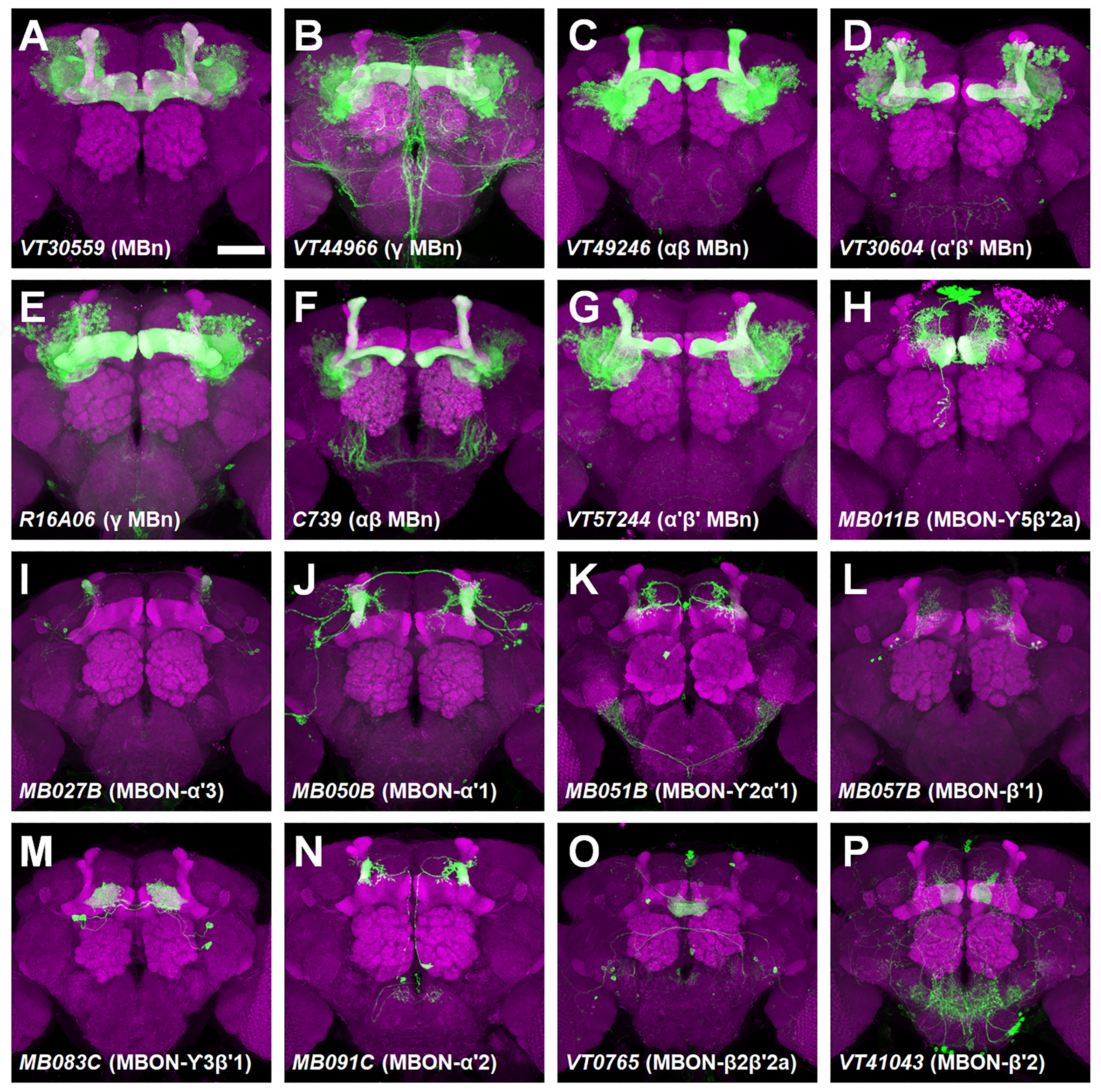

Supplement: S3 Fig — (A) MBn with VT30559-GAL4 expression. (B) γ MBn with VT44966-GAL4 expression. (C) αβ MBn with VT49246-GAL4 expression. (D) α′β′ MBn with VT30604-GAL4 expression. (E) γ MBn with R16A06-GAL4 expression. (F) αβ MBn with C739-GAL4 expression. (G) α′β′ MBn with VT57244-GAL4 expression. (H) MBON-γ5β′2a with MB011B-GAL4 expression. (I) MBON-α′3 with MB027B-GAL4 expression. (J) MBON-α′1 with MB050B-GAL4 expression. (K) MBON-γ2α′1 with MB051B-GAL4 expression. (L) MBON-β′1 with MB057B-GAL4 expression. (M) MBON-γ3β′1 with MB083C-GAL4 expression. (N) MBON-α′2 with MB091C-GAL4 expression. (O) MBON-β2β′2a with VT0765-GAL4 expression. (P) MBON-β′2 with VT41043-GAL4 expression. Each GAL4 line was crossed with the UAS-mCD8::GFP; UAS-mCD8::GFP reporter line and confocal brain imaging was performed on the progeny. Brain neuropils were counterstained with anti-DLG antibody (magenta). Scale bar, 50 μm. (TIF) [file pbio.3002332.s003.tif]

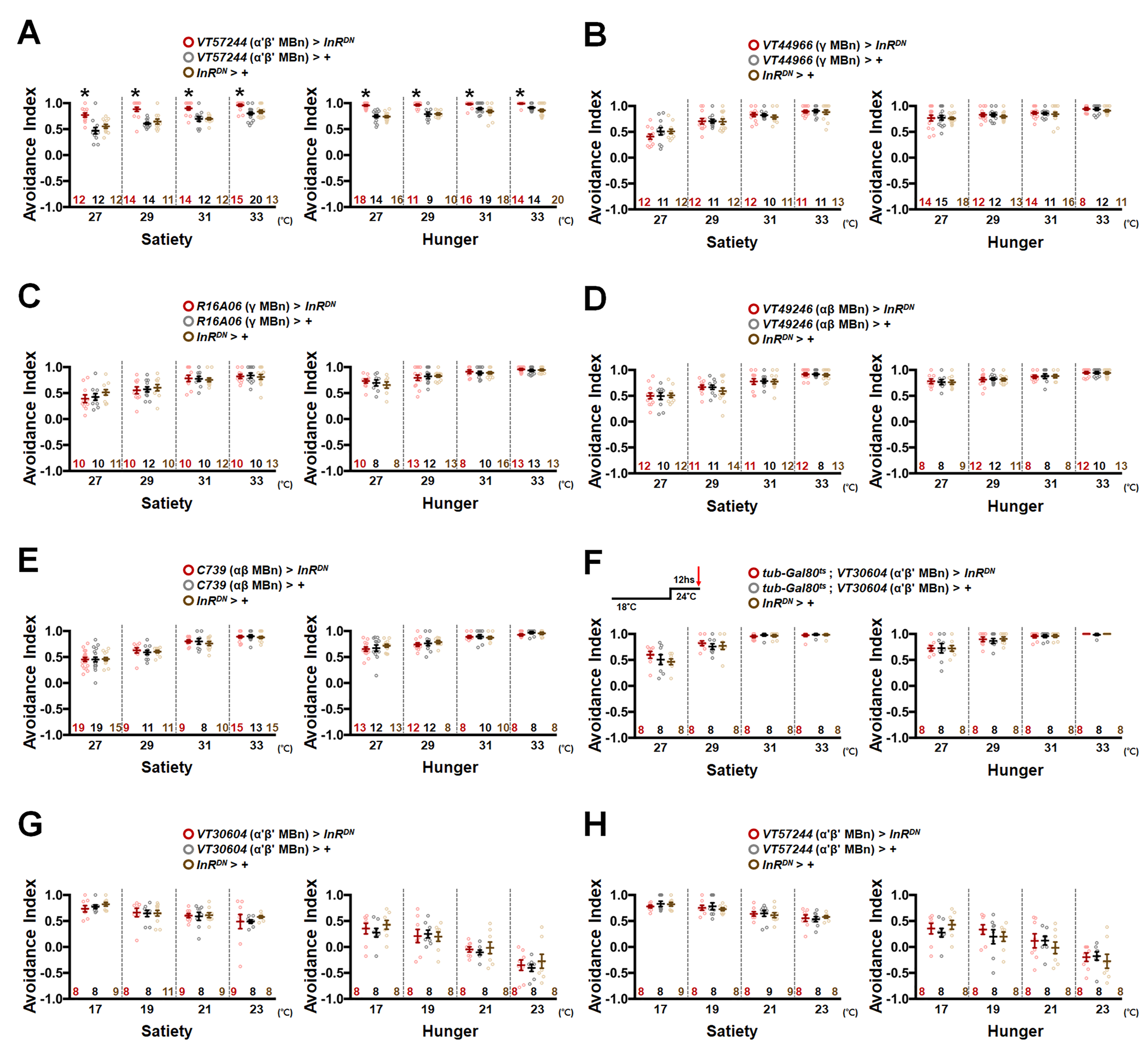

Supplement: S4 Fig — (A) Genetic expression of InRDN in α′β′ MBn via VT57244-GAL4 > UAS-InRDN increased HAB during both sated and hungry states (Satiety: P-values: 0.0003, <0.0001, <0.0001, and 0.0003 from left to right; Hunger: P-values: <0.0001, <0.0001, <0.0001, and 0.0002 from left to right). (B) Genetic expression of InRDN in γ MBn via VT44966-GAL4 > UAS-InRDN had no effect on HAB during both sated and hungry states (Satiety: P-values: 0.3221, 0.9858, 0.6006, and 0.8822 from left to right; Hunger: P-values: 0.9897, 0.6707, 0.8446, and 0.5868 from left to right). (C) Genetic expression of InRDN in γ MBn via R16A06-GAL4 > UAS-InRDN had no effect on HAB during both sated and hungry states (Satiety: P-values: 0.3765, 0.8375, 0.9045, and 0.9183 from left to right; Hunger: P-values: 0.5816, 0.8305, 0.8359, and 0.7554 from left to right). (D) Genetic expression of InRDN in αβ MBn via VT49246-GAL4 > UAS-InRDN had no effect on HAB during both sated and hungry states (Satiety: P-values: 0.9815, 0.4656, 0.9837, and 0.9286 from left to right; Hunger: P-values: 0.9478, 0.9479, 0.9743, and 0.9928 from left to right). (E) Genetic expression of InRDN in αβ MBn via C739-GAL4 > UAS-InRDN had no effect on HAB during both sated and hungry states (Satiety: P-values: 0.9846, 0.7737, 0.7182, and 0.7518 from left to right; Hunger: P-values: 0.5773, 0.6921, 0.853, and 0.4112 from left to right). (F) Permissive temperature control of Fig 2E. At permissive temperatures, there were no significant differences in HAB between tub-GAL80ts; VT30604-GAL4 > InRDN, tub-GAL80ts; VT30604-GAL4 >+, and UAS-InRDN >+ flies during sated and hungry states (Satiety: P-values: 0.4415, 0.7193, 0.7097, and 0.9129 from left to right; Hunger: P-values: 0.9994, 0.7927, 0.9944, and 0.3847 from left to right). (G) Genetic expression of InRDN in α′β′ MBn via VT30604-GAL4 > UAS-InRDN had no effect on the cold avoidance behavior during sated and hungry states (Satiety: P-values: 0.4082, 0.9822, 0.9626, and 0.7241 from left to right; Hun [file pbio.3002332.s004.tif]

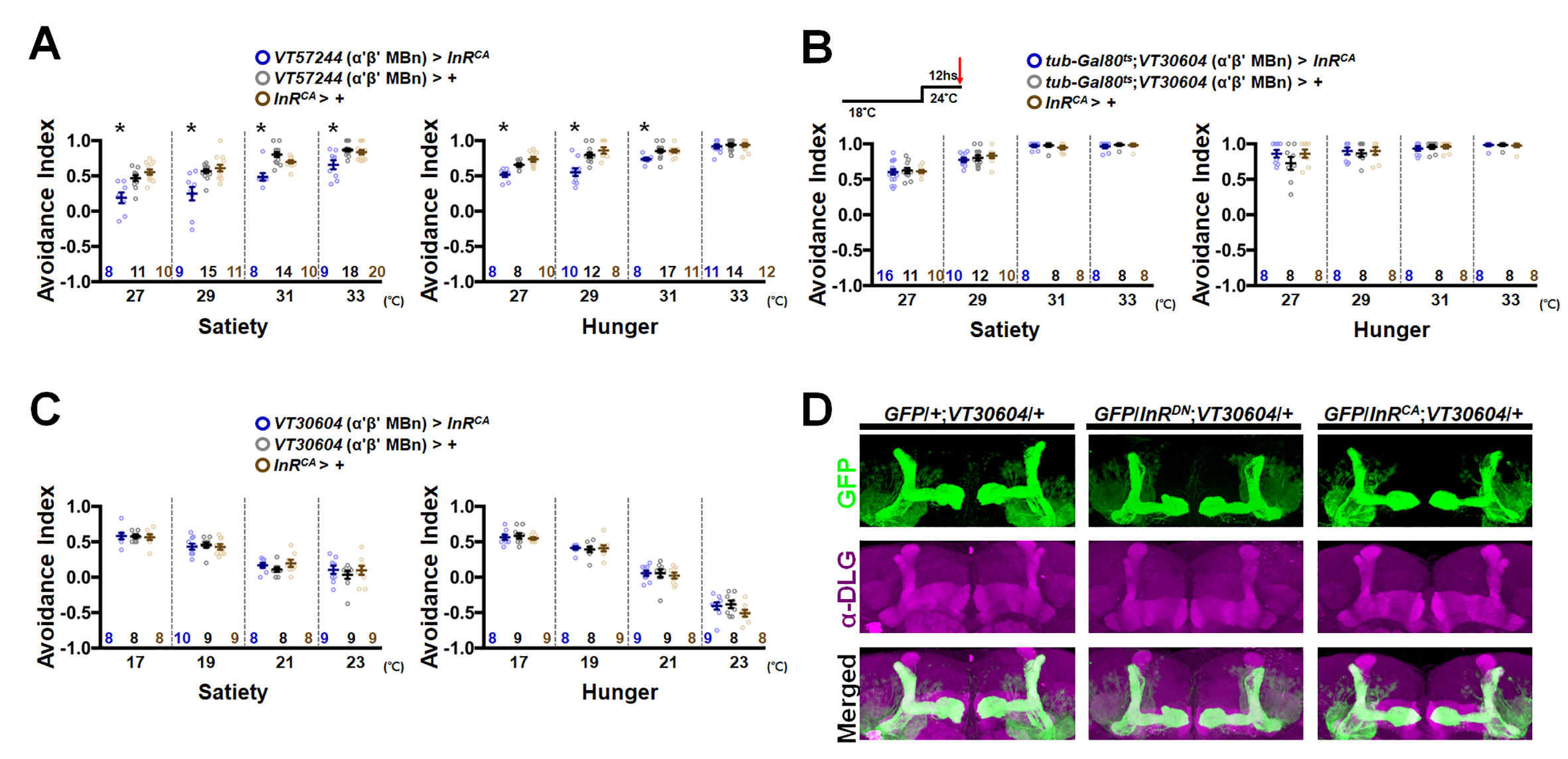

Supplement: S5 Fig — (A) Genetic expression of InRCA in α′β′ MBn via VT57244-GAL4 > UAS-InRCA decreased HAB during both sated and hungry states (Satiety: P-values: 0.0006, <0.0001, <0.0001, and <0.0001 from left to right; Hunger: P-values: 0.0002, <0.0001, 0.0039, and 0.8281 from left to right). (B) Permissive temperature control of Fig 2F. At permissive temperatures, there were no significant differences in HAB between tub-GAL80ts; VT30604-GAL4 > InRCA, tub-GAL80ts; VT30604-GAL4 >+, and UAS-InRCA >+ flies during both sated and hungry states (Satiety: P-values: 0.919, 0.503, 0.5766, and 0.6841 from left to right; Hunger: P-values: 0.2961, 0.8591, 0.7777, and 0.9495 from left to right). (C) Genetic expression of InRCA in α′β′ MBn did not affect cold avoidance behavior in both satiety and hungry states (Satiety: P-values: 0.9561, 0.8529, 0.2998, and 0.655 from left to right; Hunger: P-values: 0.7102, 0.9054, 0.843, and 0.2119 from left to right). (D) The morphology of α′β′ MBn, labeled by GFP (green), was not affected in flies carrying UAS-mCD8::GFP/UAS-InRDN; VT30604-GAL4/+ or UAS-mCD8::GFP/UAS-InRCA; VT30604-GAL4/+. Each N represents a group of 15 flies analyzed together in the behavioral assay (A–C). The data underlying this figure can be found in S1 Data. Data are represented as mean ± SEM with dots representing individual values and analyzed by one-way ANOVA followed by Tukey’s test, *P < 0.05. (TIF) [file pbio.3002332.s005.tif]

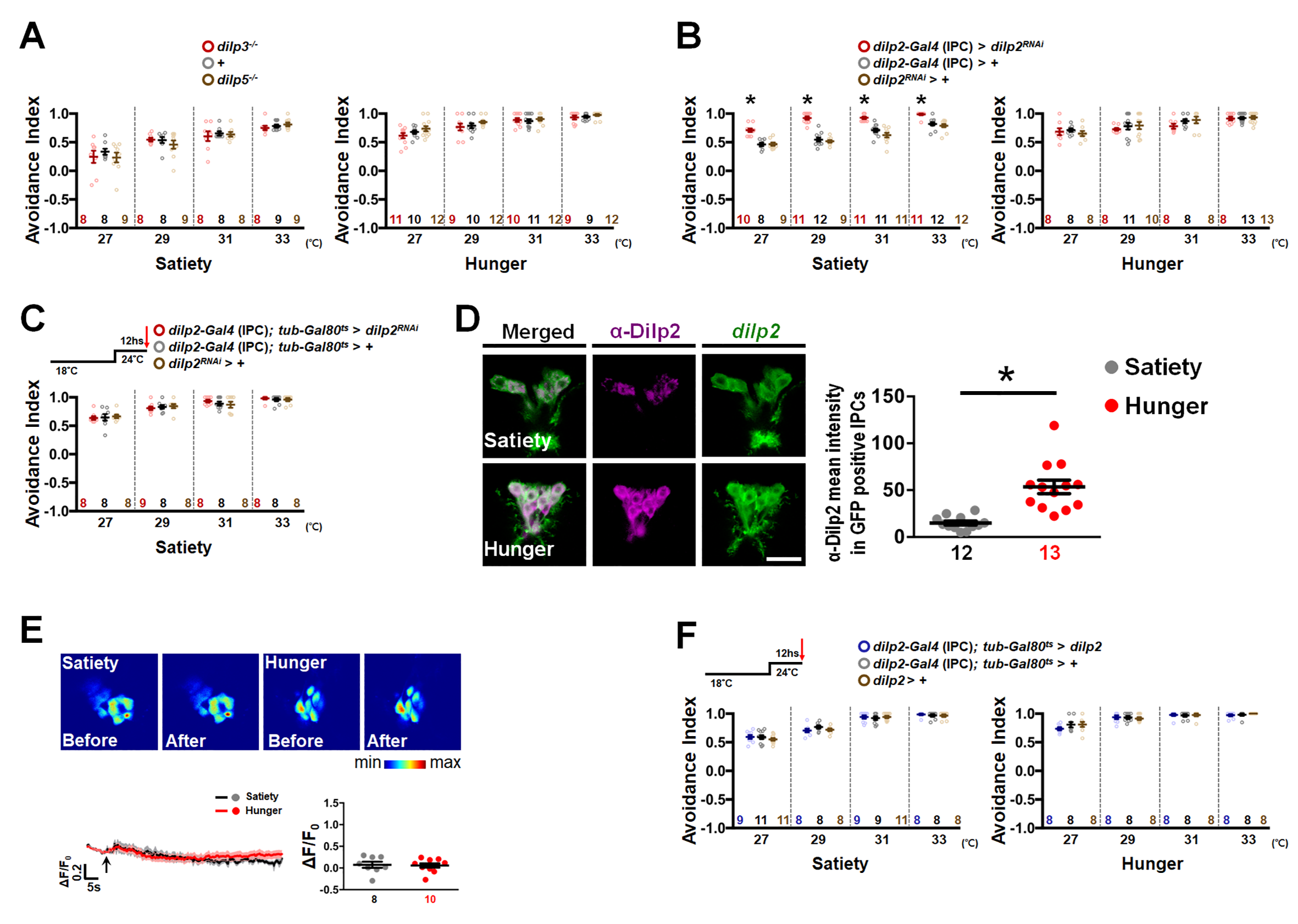

Supplement: S6 Fig — (A) HAB was not affected in dilp3 mutant flies (dilp3-/-) and dilp5 mutant flies (dilp5-/-) (Satiety: P-values: 0.6672, 0.4866, 0.8345, and 0.4314 from left to right; Hunger: P-values: 0.1133, 0.2915, 0.718, and 0.426 from left to right). (B) dilp2-GAL4 > UAS-dilp2RNAi flies exhibited an increased HAB during the sated state (Satiety: P-values: <0.0001, <0.0001, <0.0001, and <0.0001 from left to right; Hunger: P-values: 0.6612, 0.7088, 0.2353, and 0.8943 from left to right). (C) Permissive temperature control of Fig 3C. At permissive temperatures, there were no significant differences in HAB between dilp2-GAL4; tub-GAL80ts > UAS-dilp2RNAi, dilp2-GAL4; tub-GAL80ts > +, and UAS-dilp2RNAi > + flies during the sated state (Satiety: P-values: 0.8918, 0.7176, 0.5073, and 0.7762 from left to right). (D) Immunostaining with anti-Dilp2 antibody in dilp2-GAL4 > UAS-mCD8::GFP flies (left panel). Quantification of anti-Dilp2 antibody immunostaining intensity in GFP positive IPCs during sated and hungry states (right panel). The anti-Dilp2 immunostaining signals in IPCs were normalized to the signals in fan-shaped body (P <0.0001). Scale bar, 20 μm. (E) Hot stimulus did not induce the calcium response in IPCs during both feeding states. The soma of IPCs were recorded and analyzed. There were no significant differences in GCaMP intensity in IPCs before and after hot stimuli (P-values: 0.8381). The arrow under calcium response curve indicates the time points at which the hot stimuli were applied. (F) Permissive temperature control of Fig 3E. At permissive temperatures, there were no significant differences in HAB between dilp2-GAL4; tub-GAL80ts > UAS-dilp2, dilp2-GAL4; tub-GAL80ts > +, and UAS-dilp2 > + flies during sated and hungry states (Satiety: P-values: 0.5363, 0.3002, 0.8416, and 0.7235 from left to right; Hunger: P-values: 0.3413, 0.8279, 0.9225, and 0.4409 from left to right). Each N represents either a group of 15 flies analyzed together in the behavioral assay (A, B, C, [file pbio.3002332.s006.tif]

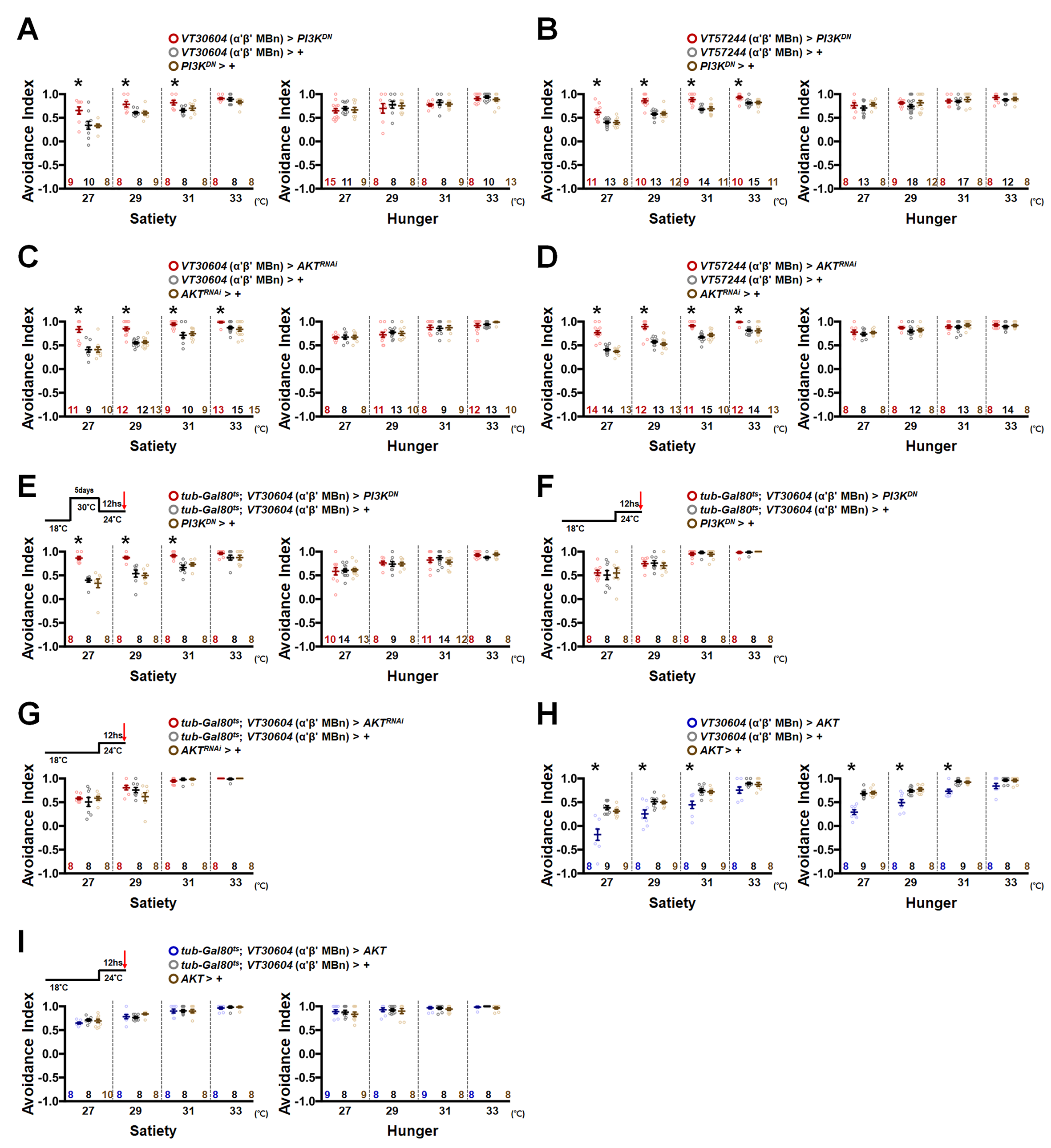

Supplement: S7 Fig — (A) Genetic expression of PI3KDN in α′β′ MBn via VT30604-GAL4 > UAS-PI3KDN increased HAB during the sated state (Satiety: P-values: 0.0055, 0.0072, 0.0341, and 0.2073 from left to right; Hunger: P-values: 0.6698, 0.7836, 0.6583, and 0.5004 from left to right). (B) Genetic expression of PI3KDN in α′β′ MBn via VT57244-GAL4 > UAS-PI3KDN increased HAB during the sated state (Satiety: P-values: 0.0002, <0.0001, <0.0001, and 0.002 from left to right; Hunger: P-values: 0.4156, 0.3309, 0.6178, and 0.5198 from left to right). (C) RNAi-mediated knockdown of AKT in α′β′ MBn via VT30604-GAL4 > UAS-AKTRNAi increased HAB during the sated state (Satiety: P-values: <0.0001, <0.0001, 0.002, and 0.0018 from left to right; Hunger: P-values: 0.9554, 0.6714, 0.9494, and 0.1951 from left to right). (D) RNAi-mediated knockdown of AKT in α′β′ MBn via VT57244-GAL4 > UAS-AKTRNAi increased HAB during the sated state (Satiety: P-values: <0.0001, <0.0001, <0.0001, and <0.0001 from left to right; Hunger: P-values: 0.6866, 0.3042, 0.5841, and 0.588 from left to right). (E) Adult-stage-specific expression of PI3KDN in α′β′ MBn via tub-GAL80ts; VT30604-GAL4 > UAS-PI3KDN increased HAB during the sated state (Satiety: P-values: <0.0001, <0.0001, 0.0003, and 0.2743 from left to right; Hunger: P-values: 0.9187, 0.9098, 0.268, and 0.1859 from left to right). (F) At permissive temperatures, there were no significant differences in HAB between tub-GAL80ts; VT30604-GAL4 > UAS-PI3KDN, tub-GAL80ts; VT30604-GAL4 > +, and UAS-PI3KDN > + flies during the sated state (Satiety: P-values: 0.9063, 0.7536, 0.7213, and 0.5989 from left to right). (G) Permissive temperature control of Fig 3G. At permissive temperatures, there were no significant differences in HAB between tub-GAL80ts; VT30604-GAL4 > UAS-AKTRNAi, tub-GAL80ts; VT30604-GAL4 > +, and UAS-AKTRNAi > + flies during the sated state (Satiety: P-values: 0.5946, 0.1219, 0.4254, and 0.3847 from left to right). (H) Genetic expression of AKT in α′β′ MBn via VT30604 [file pbio.3002332.s007.tif]

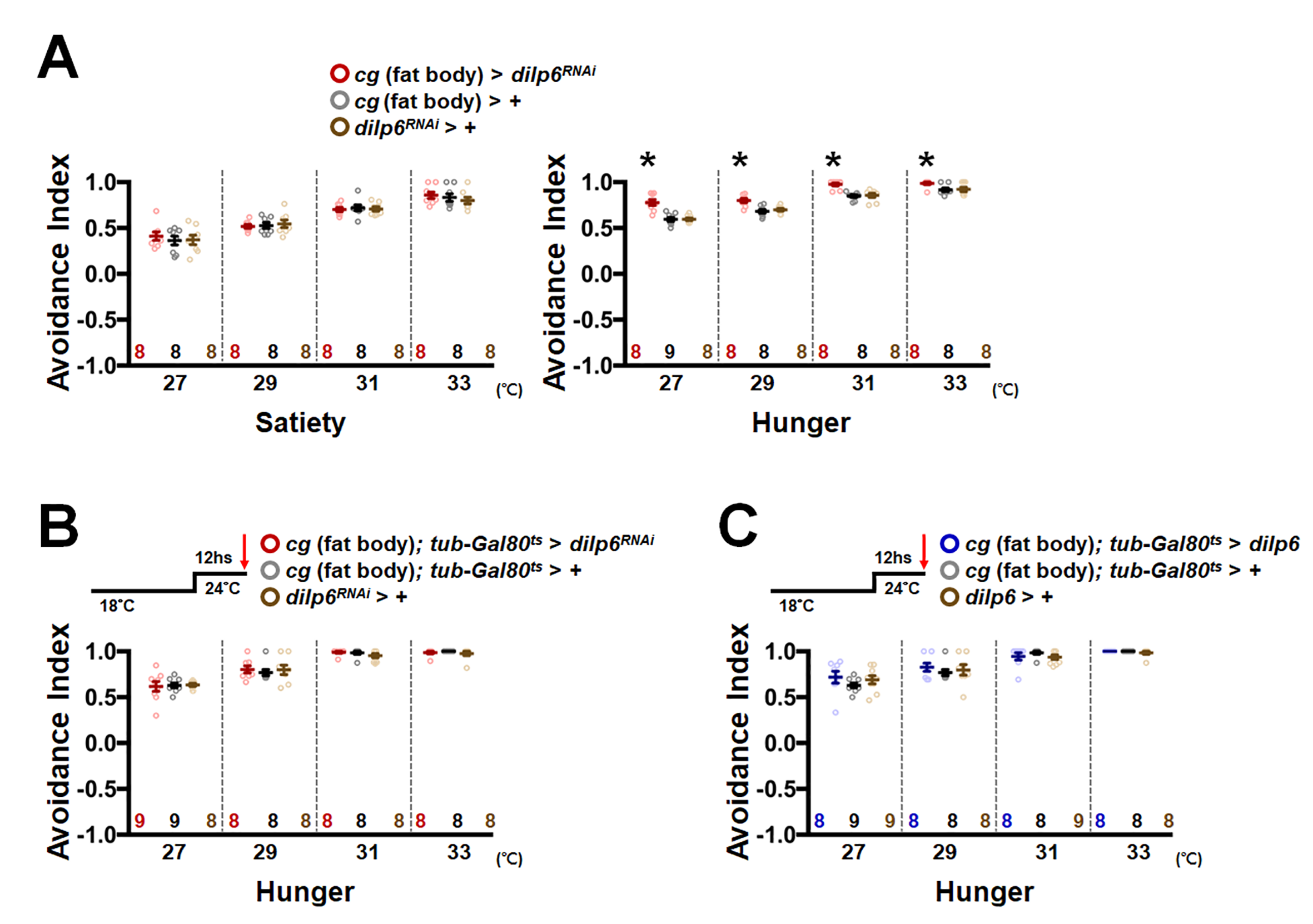

Supplement: S8 Fig — (A) Genetic knockdown of dilp6 in the fat body via cg-GAL4 > UAS-dilp6RNAi increased HAB during the hungry state (Satiety: P-values: 0.746, 0.8274, 0.8812, and 0.5568 from left to right; Hunger: P-values: <0.0001, 0.0007, <0.0001, and 0.0338 from left to right). (B) Permissive temperature control of Fig 4C. At permissive temperatures, there were no significant differences in HAB between cg-GAL4; tub-GAL80ts > UAS-dilp6RNAi, cg-GAL4; tub-GAL80ts >+, and UAS-dilp6RNAi > + flies during the hungry state (Hunger: P-values: 0.9535, 0.8192, 0.305, and 0.576 from left to right). (C) Permissive temperature control of Fig 4E. At permissive temperatures, there were no significant differences in HAB between cg-GAL4; tub-GAL80ts > UAS-dilp6, cg-GAL4; tub-GAL80ts >+, and UAS-dilp6 > + flies during the hungry state (Hunger: P-values: 0.3979, 0.6643, 0.475, and 0.3847 from left to right). Each N represents a group of 15 flies analyzed together in the behavioral assay. Data are represented as mean ± SEM with dots representing individual values. The data underlying this figure can be found in S1 Data. Data were analyzed by one-way ANOVA followed by Tukey’s test. *P < 0.05. (TIF) [file pbio.3002332.s008.tif]

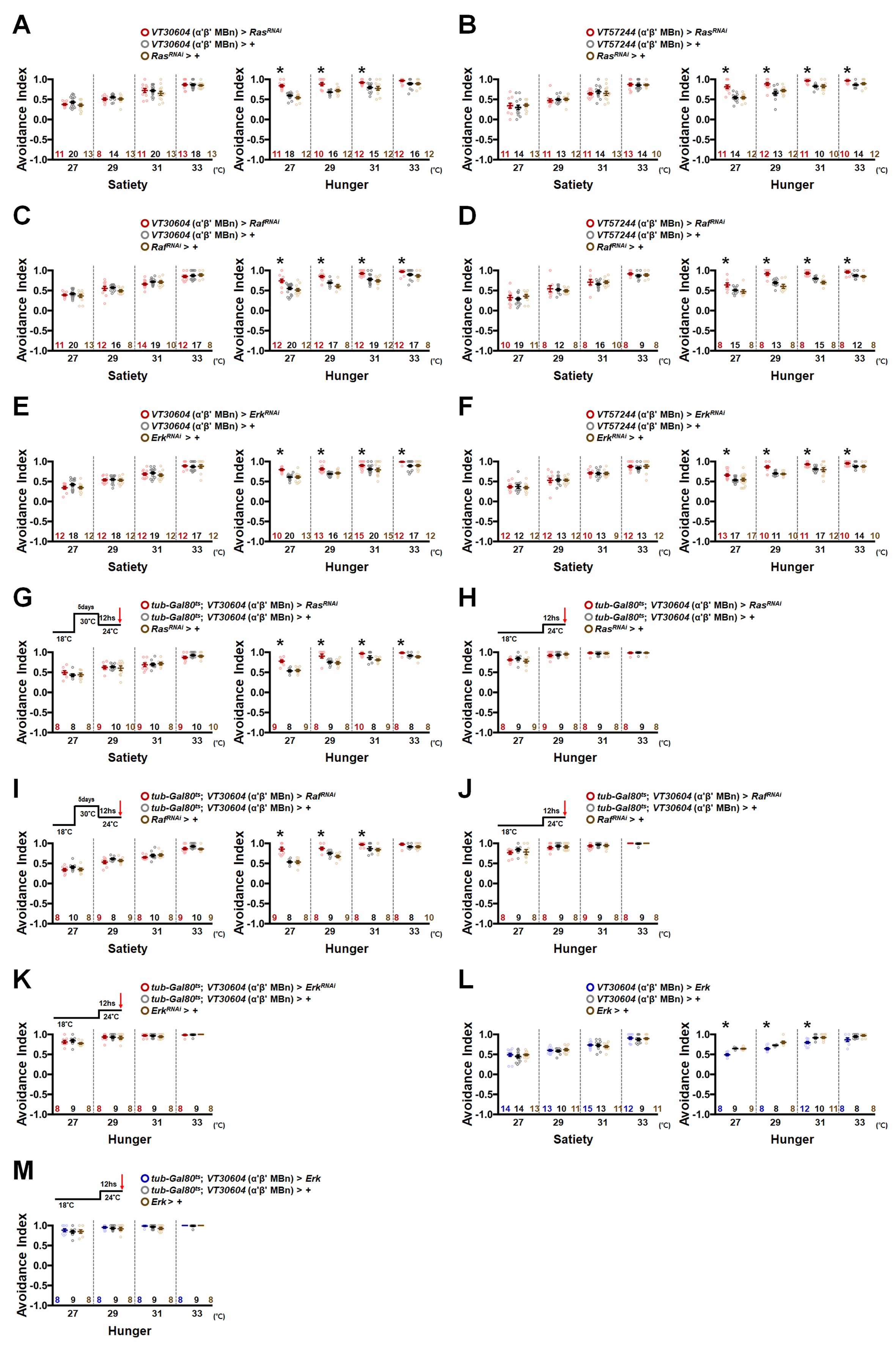

Supplement: S9 Fig — (A) Genetic knockdown of Ras in α′β′ MBn via VT30604-GAL4 > UAS-RasRNAi increased HAB during the hungry state (Satiety: P-values: 0.1516, 0.2869, 0.4018, and 0.8412 from left to right; Hunger: P-values: <0.0001, <0.0001, 0.0107, and 0.0599 from left to right). (B) Genetic knockdown of Ras in α′β′ MBn via VT57244-GAL4 > UAS-RasRNAi increased HAB during the hungry state (Satiety: P-values: 0.7096, 0.7749, 0.7044, and 0.8978 from left to right; Hunger: P-values: <0.0001, 0.0002, 0.0006, and 0.0031 from left to right). (C) Genetic knockdown of Raf in α′β′ MBn via VT30604-GAL4 > UAS-RafRNAi increased HAB during the hungry state (Satiety: P-values: 0.7734, 0.2772, 0.197, and 0.6603 from left to right; Hunger: P-values: 0.0002, <0.0001, 0.0001, and 0.0356 from left to right). (D) Genetic knockdown of Raf in α′β′ MBn via VT57244-GAL4 > UAS-RafRNAi increased HAB during the hungry state (Satiety: P-values: 0.4945, 0.7428, 0.5287, and 0.5035 from left to right; Hunger: P-values: 0.0099, <0.0001, <0.0001, and 0.0111 from left to right). (E) Genetic knockdown of Erk in α′β′ MBn via VT30604-GAL4 > UAS-ErkRNAi increased HAB during the hungry state (Satiety: P-values: 0.3408, 0.8722, 0.3893, and 0.8622 from left to right; Hunger: P-values: <0.0001, 0.0003, 0.0298, and 0.0081 from left to right). (F) Genetic knockdown of Erk in α′β′ MBn via VT57244-GAL4 > UAS-ErkRNAi increased HAB during the hungry state (Satiety: P-values: 0.845, 0.9818, 0.9571, and 0.5833 from left to right; Hunger: P-values: 0.0133, <0.0001, 0.0064, and 0.0386 from left to right). (G) Adult-stage-specific knockdown of Ras in α′β′ MBn via tub-GAL80ts; VT30604-GAL4 > UAS-RasRNAi increased HAB during the hungry state (Satiety: P-values: 0.4816, 0.8399, 0.8754, and 0.2618 from left to right; Hunger: P-values: <0.0001, 0.0058, 0.0011, and 0.0091 from left to right). (H) At permissive temperatures, there were no significant differences in HAB between tub-GAL80ts; VT30604-GAL4 > UAS-RasRNAi, tub-GAL80ts; VT30604-GAL4 > [file pbio.3002332.s009.tif]

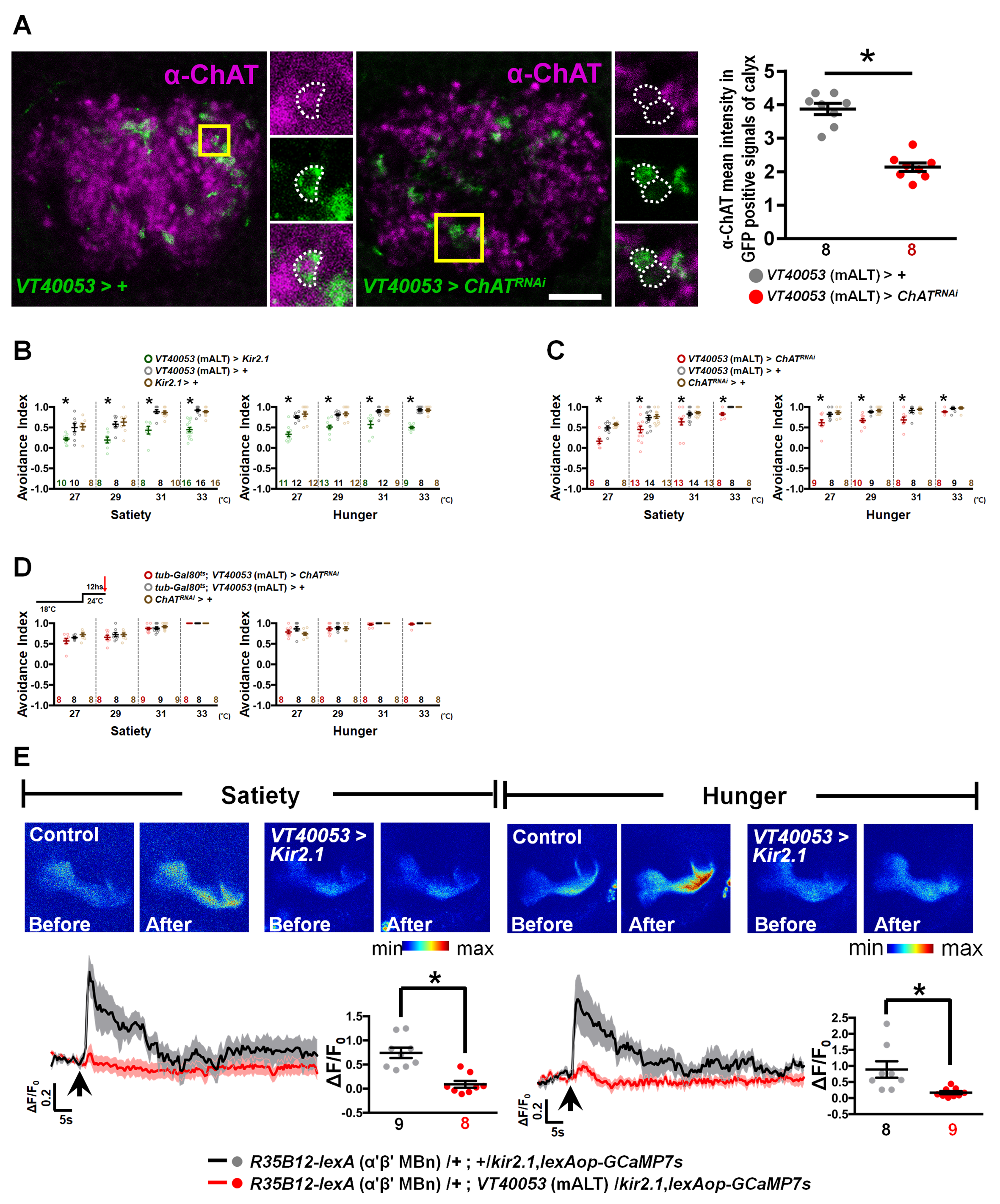

Supplement: S10 Fig — (A) Immunostaining with ChAT antibody in UAS-mCD8::GFP; VT40053-GAL4 > + (VT40053 > +) and UAS-mCD8::GFP; VT40053-GAL4 > UAS-ChATRNAi (VT40053 > ChATRNAi) flies (left panel). Quantification of anti-ChAT antibody immunostaining intensity in GFP positive signals of the calyx (right panel). The anti-ChAT immunostaining signals in the calyx were normalized to the signals in the peduncle (P < 0.0001). Scale bar, 10 μm. (B) Silencing mALT neuronal activity via Kir2.1 expression (VT40053-GAL4 > UAS-kir2.1) decreased HAB during both sated and hungry states (Satiety: P-values: 0.0063, 0.0008, <0.0001, and <0.0001 from left to right; Hunger: P-values: <0.0001, <0.0001, <0.0001, and <0.0001 from left to right). (C) Genetic knockdown of ChAT in mALT neurons (VT40053-GAL4 > UAS-ChATRNAi) decreased HAB during both sated and hungry states (Satiety: P-values: <0.0001, 0.0015, 0.0059, and <0.0001 from left to right; Hunger: P-values: 0.0091, <0.0001, 0.0037, and 0.0222 from left to right). (D) Permissive temperature control of Fig 6E. At permissive temperatures, there were no significant differences in HAB between tub-GAL80ts; VT40053-GAL4 > UAS-ChATRNAi, tub-GAL80ts; VT40053-GAL4 > + and UAS-ChATRNAi > + flies during both feeding states (Satiety: P-values: 0.0676, 0.4831, 0.524, and 1 from left to right; Hunger: P-values: 0.1797, 0.9068, 0.1219, and 0.3847 from left to right). (E) Hot stimulus induced calcium responses in α′β′ MBn (black), while silencing mALT activity via Kir2.1 expression inhibited hot-induced calcium responses in α′β′ MBn (red) (P = 0.0002 for satiety; P = 0.0101 for hunger). The GCaMP intensity changes (ΔF/F0) in MB β′ lobe were recorded and analyzed. The arrows under each calcium response curve indicate the time points at which the hot stimulus was applied. Each N represents a single fly in ChAT immunostaining experiments (A) and calcium imaging experiments (E) or group of 15 flies analyzed together in the behavioral assay (B–D). Data are represented as mean ± [file pbio.3002332.s010.tif]

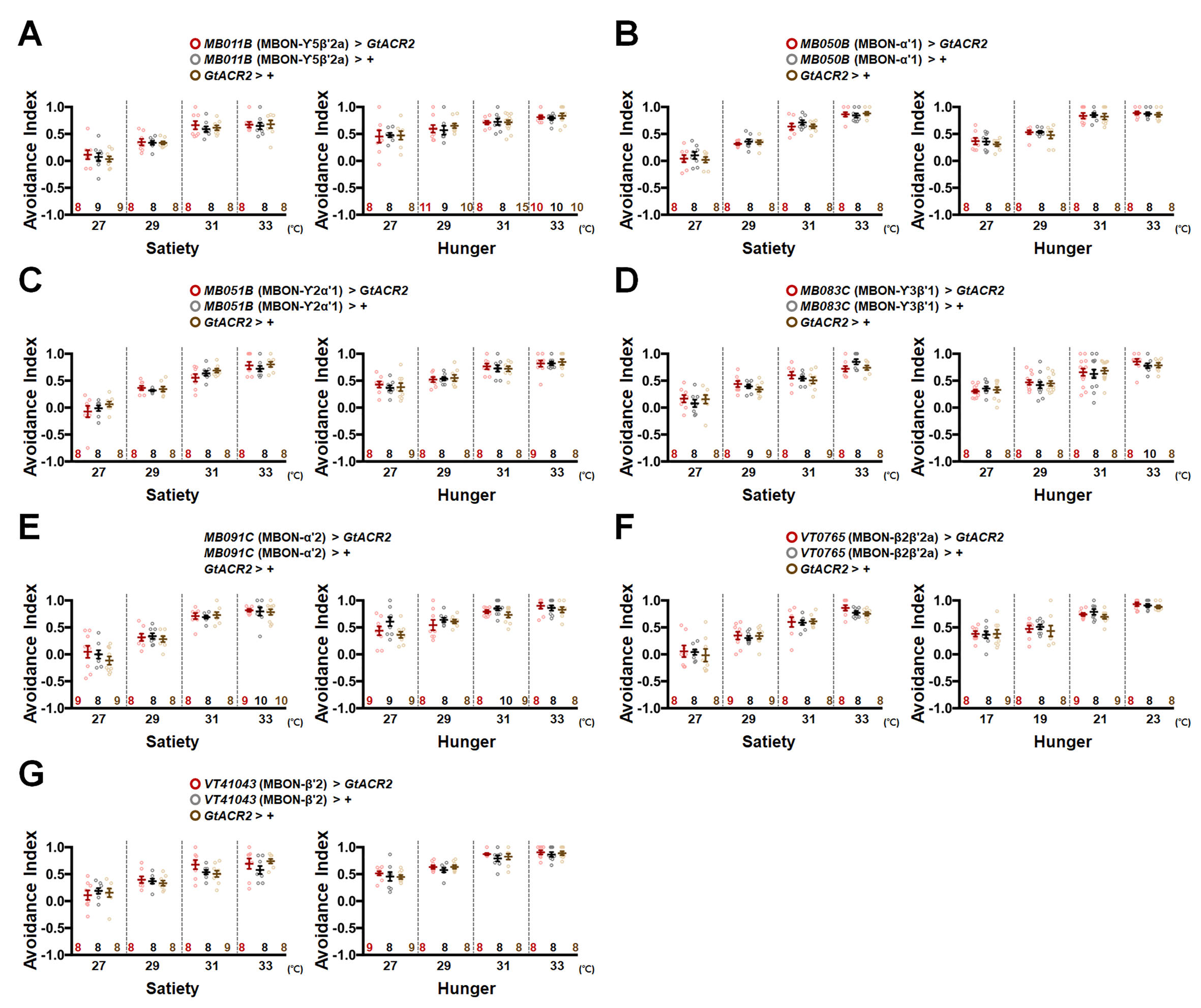

Supplement: S11 Fig — (A) Optogenetic silencing of MBON-γ5β′2a activity via GtACR2 expression (MB011B-GAL4 > UAS-GtACR2) had no effect on HAB during both sated and hungry states (Satiety: P-values: 0.7164, 0.971, 0.6755, and 0.9396 from left to right; Hunger: P-values: 0.9746, 0.9884, 0.9782, and 0.867 from left to right). (B) Optogenetic silencing of MBON-α′1 activity via GtACR2 expression (MB050B-GAL4 > UAS-GtACR2) had no effect on HAB during both sated and hungry states (Satiety: P-values: 0.6302, 0.6589, 0.4405, and 0.7501 from left to right; Hunger: P-values: 0.6951, 0.5973, 0.9209, and 0.7727 from left to right). (C) Optogenetic silencing of MBON-γ2α′1 activity via GtACR2 expression (MB051B-GAL4 > UAS-GtACR2) had no effect on HAB during both sated and hungry states (Satiety: P-values: 0.4358, 0.6369, 0.2102, and 0.5801, from left to right; Hunger: P-values: 0.7697, 0.903, 0.8262, and 0.9061, from left to right). (D) Optogenetic silencing of MBON-γ3β′1 activity via GtACR2 expression (MB083C-GAL4 > UAS-GtACR2) had no effect on HAB during both sated and hungry states (Satiety: P-values: 0.657, 0.2801, 0.4716, and 0.1395, from left to right; Hunger: P-values: 0.9009, 0.6699, 0.7454, and 0.9781 from left to right). (E) Optogenetic silencing of MBON-α′2 activity via GtACR2 expression (MB091C-GAL4 > UAS-GtACR2) had no effect on HAB during both sated and hungry states (Satiety: P-values: 0.4278, 0.7957, 0.8411, and 0.6193 from left to right; Hunger: P-values: 0.0823, 0.5552, 0.1326, and 0.602, from left to right). (F) Optogenetic silencing of MBON-β2β′2a activity via GtACR2 expression (VT0765-GAL4 > UAS-GtACR2) had no effect on HAB during both sated and hungry states (Satiety: P-values: 0.8509, 0.8318, 0.9641, and 0.1453 from left to right; Hunger: P-values: 0.9756, 0.7681, 0.2891, and 0.5207, from left to right). (G) Optogenetic silencing of MBON-β′2 activity via GtACR2 expression (VT41043-GAL4 > UAS-GtACR2) had no effect on HAB during both sated and hungry states (Satiety: P-values: 0.75 [file pbio.3002332.s011.tif]

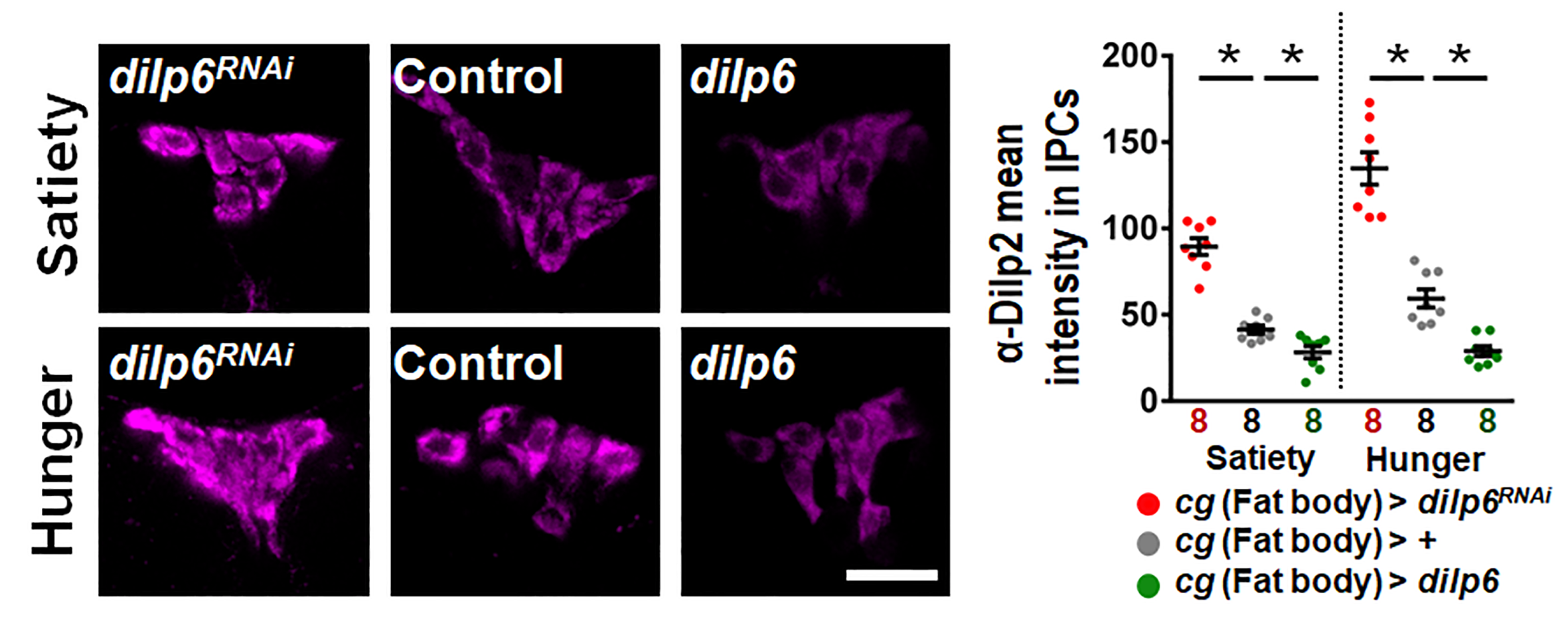

Supplement: S12 Fig — Immunostaining with anti-Dilp2 antibody in cg-GAL4 > UAS-dilp6RNAi (dilp6RNAi), cg-GAL4 > + (Control), and cg-GAL4 > UAS-dilp6 (dilp6) flies (left panel). Quantification of anti-Dilp2 immunopositive signals in IPC during sated and hungry states (right panel). The anti-Dilp2 immunostaining signals in IPCs were normalized to the signals in the fan-shaped body (Satiety: P-values: <0.0001 and 0.0077 from left to right; Hunger: P-values: <0.0001 and 0.0002 from left to right). Scale bar, 20 μm. Each N represents a single fly in Dilp2 immunostaining experiments. Data are represented as mean ± SEM with dots representing individual values. The data underlying this figure can be found in S1 Data. Data were analyzed by unpaired two-tailed t test. *P < 0.05. (TIF) [file pbio.3002332.s012.tif]

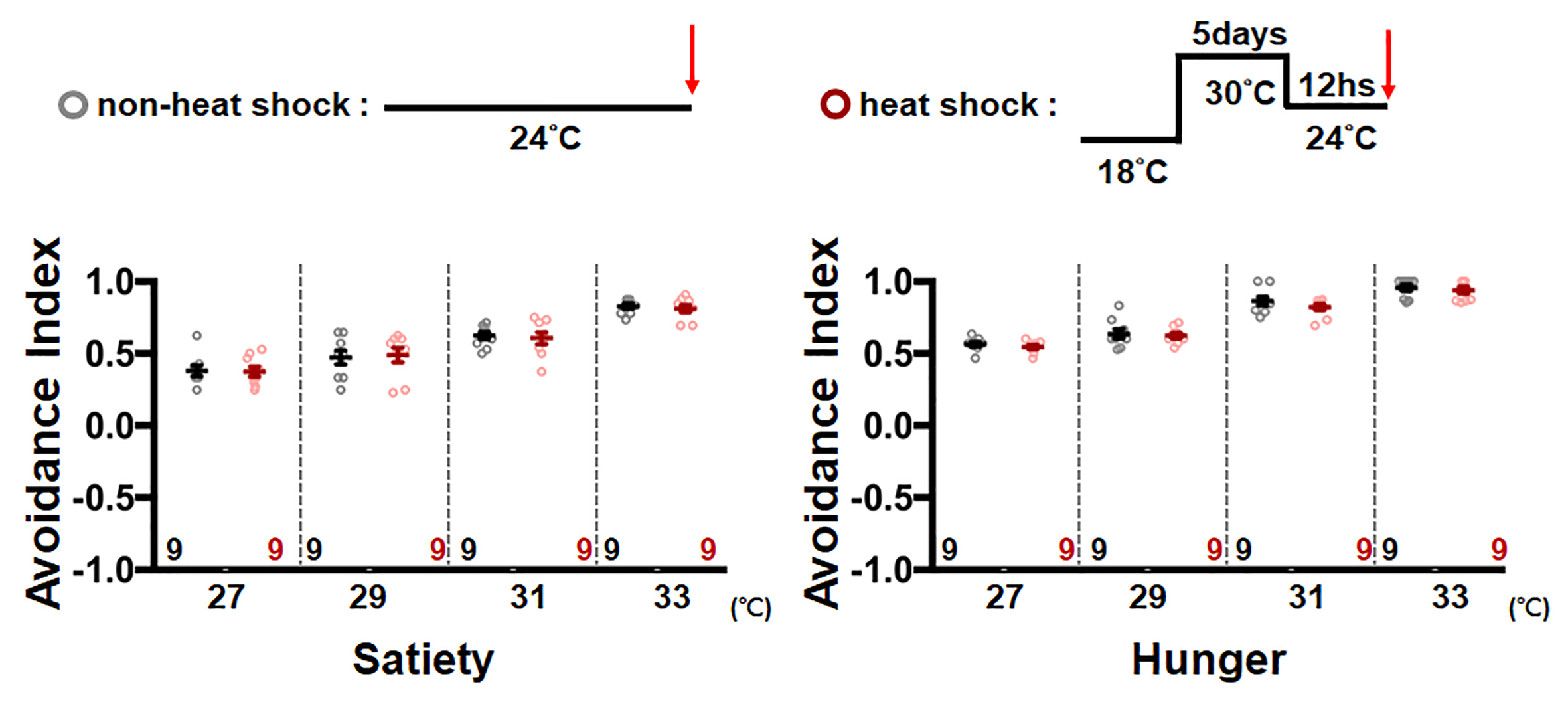

Supplement: S13 Fig — Wild-type flies were raised under a constant temperature of either 24°C (non-heat shock) or 18°C during embryonic and larval development, transferred to 30°C for 5 days after adult eclosion, and then shifted back to 24°C for 12 h before the HAB assay was conducted (heat shock). There were no significant differences in HAB between non-heat shock and heat shock groups in both feeding states (Satiety: P-values: 0.9241, 0.8008, 0.7063, and 0.5931 from left to right; Hunger: P-values: 0.3508, 0.7399, 0.2583, and 0.6540 from left to right). Each N represents a group of 15 flies analyzed together in the behavioral assay. Data are represented as mean ± SEM with dots representing individual values. The data underlying this figure can be found in S1 Data. Data were analyzed by unpaired two-tailed t test. (TIF) [file pbio.3002332.s013.tif]
