# Supplementary material for: Independent insulin signaling modulators govern hot avoidance under different feeding states
Source: PLoS Biol. 2023 Oct 17;21(10):e3002332. doi: 10.1371/journal.pbio.3002332 (PMC10581474; doi:10.1371/journal.pbio.3002332)
Supplement: Attachment — Submitted filename: Response to Reviewers.docx [file pbio.3002332.s016.docx]

**Reviewer #1:** This manuscript uses behavioral assays and calcium imaging to understand how fed status affects hot avoidance behavior (HAB). The authors provide compelling evidence to support their findings. The authors first show hungry flies exhibit stronger HAB than fed flies. Then, they demonstrate different Dilps inhibit α'β' MBns in different fed statuses. Finally, they identify the neural circuits that control HAB from mALT projection neurons to α'β' MBns to MBONs. Experiments were carefully designed, and the manuscript was well-written. I enjoyed reading this manuscript. The following are two comments to help improve the manuscript.

1.	Besides α'β', αβ and γ MBns are also necessary for HAB under satiety conditions (Fig 1E, F). How does fed status regulate αβ and γ MBns to regulate HAB in satiety conditions?

Author’s response: Dopaminergic neurons regulate the feeding state and MBn activity in *Drosophila* (Tsao CH et al., 2018, Siju KP et al., 2020, Yamagata N et al., 2016). The dopaminergic PPL1-α'2α2 neurons only receive satiety signals whereas PAM-β2β'2a and PPL1-γ2α'1 receive both satiety and hunger signals (Tsao CH et al., 2018). Additionally, nutritious sugar immediately suppresses the dopaminergic PAM-γ3 activity which provides a positive reinforcing signal for sugar-reward memory formation (Yamagata N et al., 2016). We, therefore, postulate that αβ and γ MBn activity is regulated by dopaminergic neurons that contribute to proper HAB in satiety conditions. We have added this information to the discussion section of the revised manuscript (Lines: 664–686).

2.	Considering that the inputs from mALT are similar in hungry and satiety conditions (Fig 5F), it is valuable to demonstrate whether the α'β' responses are also similar in both conditions in the absence of Dilp regulation. 
Author’s response: Thank you for your constructive comments. We performed live brain calcium imaging experiments under *InR^DN^* expressing conditions in α'β' MBn and found no significant differences in hot responses in α'β' MBn (Fig 6H). Moreover, the hot responses in α'β' MBn in *dilp2^-/-^* and *dilp6^LOF^* double mutant flies did not differ significantly (Fig 6I).

**Reviewer #2:** This study addresses the role of animal feeding status to thermosensation using Drosophila melanogaster as a model. The study shows convincingly that starved animal exhibit hot avoidance behavior compared to well fed animals. The study identified a group of mushroom body neurons that are responsible for the feeding-state dependent hot avoidance behavior. Moreover, neuros which mediate the heat input to the mushroom body neurons as well as output neurons mediating the hot avoidance behavior are characterized. The authors present evidence for a dual mode of regulation of these neurons through insulin-like receptor signaling. Insulin-like peptide (ILP) 2, secreted from the insulin-producing cell signal upon the fed state, while fat body-derived ILP6 signals the starved state. In sum, the study presents an elegant example of how insulin-like signaling, which is responsive to feeding state, interacts with animal behavior and provides a thorough characterization of the underlying neuronal mechanism. The experimental evidence presented in the study in extensive and the experiments are well controlled. Each main conclusion of the study is robustly supported by several lines of evidence. The findings are likely of broad scientific interest and therefore the manuscript is, in my opinion, suitable for Plos Biology.

I have one major concern that should be addressed prior to publication:

The authors propose a provocative model to explain their data of opposing functions of ILP2 and ILP6. The model implies that the fed state-induced signal by ILP2 is mediated through the PI3K/AKT pathway, the starvation-induced ILP6 signaling would activate the MAPK/ERK pathway. This is an elegant model, as it would explain how the Insulin-like receptor can mediate opposing signals in response to different ligands. However, to my knowledge, there is not known biochemical mechanism that would explain such ligand-specific signaling outputs. While the genetic manipulation of AKT vs ERK signaling as well as the pAKT and pERK signals upon fed and starved states, support the conclusion, the presented data does not sufficiently test the proposed model. Currently, it remains a valid possibility that ILP6 controls the mushroom body neurons indirectly.

Therefore, I request the following revisions:

1. Further experiments should be conducted to analyze the role of insulin-like signaling in the mushroom body neurons. Specifically, the authors should analyze, how pAKT and pERK signals change upon loss-of-function of InR, ILP2 and ILP6 in fed and starved animals.  If necessary, the strength of conclusions should be adjusted according to the data. 
Author’s response: Thank you for your constructive comments. We performed whole-mount brain immunostaining with anti-pAKT and anti-pERK antibodies in flies with *InR^DN^* expression in α'β' MBn, *dilp2^RNAi^* expression in IPCs, or *dilp6^RNAi^* expression in the fat body. Our results revealed that genetic expression of *InR^DN^* in α′β′ MBn ameliorated the increased levels of pAKT and pERK in sated and hungry flies (Fig 5C and 5D). Further, the genetic expression of *dilp2^RNAi^* in IPCs also ameliorated the increased level of pAKT in sated flies (Fig. 5E), whereas the genetic expression of *dilp6^RNAi^* in the fat body ameliorated the increased levels of pERK in hungry flies (Fig 5F). These results suggest that satiety-induced Dilp2 secretion by IPCs triggers intracellular signaling in α′β′ MBn, which is distinct from hunger-induced Dilp6 secretion from the fat body. We have added this information to the revised manuscript (Lines: 408–421 and 456–480).

2. Analysis of AKT and ERK phosphorylation in fed and starved states is shown in supplementary data (Figure S7J and Figure S9N). This data, as well as the additional data from the requested experiments, should be displayed in a main figure.
Author’s response: We have moved Figures S7J & S9N to the main text (Figs 5A and 5B) as suggested.

**Reviewer #3:** Chiang et al. investigate the role of the mushroom body (MB) in temperature preference and food-seeking behaviors in Drosophila. They show that hungry flies exhibit stronger avoidance of hot stimuli compared to sated flies, while cold avoidance behavior is similar. The study identifies the neurons involved in conveying hot signals and reveals that MB activity is required for hot avoidance in both feeding states. Insulin signaling and specific Dilp signals play a role in regulating hot sensation and MB neuron activity. The study also identifies downstream circuits and signaling pathways involved in executing hot avoidance behavior. Overall, the authors suggest that distinct Dilp signals mediate MB neuron activity for proper hot avoidance behavior in different feeding states.

General comments:

In general, the paper is interesting and, in my opinion, could be appropriate for publication in PlosBiology provided that several issues are successfully addressed. The topic of how the perception of an external stimulus is changed by an internal state and the behavioral consequence of this altered perception is a very relevant topic. Clearly, the authors performed a remarkable quantity of experiments and manipulations. However, I would strongly suggest to simplify and make the story easier to follow. It would, for example, help in the results session to explain in more detail the reasoning behind the experiments. Moreover, a suggestion could be to partially rephrase the paper in terms of shifting of the setpoint and switch in valence of the hot stimulus rather than strong/weak hot avoidance behavior (Figure 1 C,E,G clearly show approach for 27 and 29 degrees). It is also very difficult to compare the relative differences between hungry and satiated flies across manipulations. The scale is bigger than the average effect (0-0.5-1 vs changes of maybe 0.1-0.2). Sometimes you have to believe the authors that starved flies are still doing more HAB than satiated ones. Finally, the role of dopaminergic neurons innervating the MB is somewhat ignored in the entire paper, which I find extremely surprising given the important role of these neurons in modulating (innate) behavior due to hunger and through MB output neurons (including in particular the b'1 compartment) and their response to temperature (see Tomchik, J Neurosci 2013, Bracker et al., Current Biology 2013, Siju et al., Current Biology 2020 etc.). Moreover, plenty of literature links dopamine, insulin and temperature in different processes in humans and rodents. Thus, this should be at least discussed in the text.

*For the point of: “to partially rephrase the paper in terms of shifting of the setpoint and switch in valence of the hot stimulus rather than strong/weak hot avoidance behavior (Figure 1 C,E,G clearly show approach for 27 and 29 degrees).”*

Author’s response: We observed that temporal blue light irradiation slightly shifts the HAB in flies (see Figs 1B and S2B), which is presented in Figures 1C, 1E, and 1G. Therefore, flies showed a preference for 27°C and 29°C as mentioned by the reviewer. However, differences in HAB between sated and hungry flies were observed under blue light treatment conditions (S2B Fig). We have rephrased the statement in Lines 158–161 “Resultantly, temporal blue or red light irradiation slightly shifted the HAB (S2B and S2C Fig) in comparison with the HAB without light-irradiation (Fig 1B), indicating that exposure to light may result in a shift the set point and a switch in the valence of the hot stimulus” as suggested.

*For the point of: “The scale is bigger than the average effect (0-0.5-1 vs changes of maybe 0.1-0.2).”*

Author’s response: Thank you for the comment. We have implemented the same scale in each figure of all behavioral data throughout the manuscript. This should help readers compare the behavioral results from different experiments and avoid confusion.

*For the point of: “the role of dopaminergic neurons innervating the MB is somewhat ignored in the entire paper”.*

Author’s response: We are grateful for your constructive comments. We have added discussions regarding the roles of dopaminergic neurons innervating MBn in hunger and temperature preference modulations in the revised manuscript (Lines: 661­–686)

Specific comments:

*	Line 132: Fig S1 B-C are not addressed in the text 
Author’s response: We have cited these figures in the main text (Lines: 133–140) as suggested.

*	Line 147-148: not clear what the authors mean by temporally irradiated. In general, the protocol length and characteristics should be addressed more in detail. In the main text and methods it is stated that the total exp duration is 3 minutes and the flies were irradiated with light for the entire duration of the exp. However, in the Supplementary figure 1 E-F it is actually depicted as 3 min + 3 min (+ light)  + 3 min.
Author’s response: For optogenetic inhibition or activation, flies were irradiated during the entire duration (3 min) of the behavioral test. Figures S1E & S1F show that exposure to blue or red light has no effect on the set temperature of the aluminum plate of our device. Therefore, we performed the long-term temperature recording (9-min temperature recording with 3-min light irradiation) on the aluminum plate. We apologize for not being very clear. We have now added the following statement “Three minutes exposure to blue or red light did not alter the setting temperatures on the aluminum plates of the thermoelectric device” (Lines: 156­–158). We have also emphasized the “3 min” light irradiation treatment for our optogenetic experiments in the Materials and Methods section (Lines: 809–814).

*	Line 207: Fig. S2H: would it not be a better control to puff cold water and show no response? After all, the difference in HAB happens because starved flies avoid hot temperatures more than WT but they behave similarly to WT for cold temperatures (Figure 1 B).
Author’s response: Thank you for your comments. The main purpose of the experiment (Fig S2H) was to exclude the movement artifacts during live calcium brain imaging. At room temperature, no significant calcium responses were observed in each MBn subset, suggesting that movement artifacts can be excluded from our live calcium brain imaging experiments. We have added an explanation of the purpose of this experiment in the revised manuscript (Lines: 216–219). Cold stimuli have been demonstrated to induce significant calcium responses in MBn (Shih HW et al., 2015); therefore, we did not use cold stimuli as control.

*	Lines 217-223: maybe here the authors could clarify their reasoning in terms of expected consequences on behavior following depletion and over-expression of insulin receptors. HAB looks very different in Figure 1 C, D and Figure 2 A, B. The effects on behavior when the MB is manipulated vs InR are very different.

Author’s response: We have added a statement to clarify the reasons behind investigating InR as suggested (Lines: 230–232 and 234–235). We agree with the reviewer that HAB looks very different in Figs 1C-D and Figs 1A-B. In fact, we found that temporal blue or red light irradiation slightly shifts the HAB (Figs S2B-S2C) when compared to the HAB without light irradiation (Fig. 1B). Therefore, the HAB values are very different in Figs 1C-D and Figs 2A-B since Figures 1C-D represent results of experiments performed under light irradiation, whereas Figs 2A-B represent results of experiments under no irradiation conditions. We have added more details regarding this for clarity (Lines: 156–161).

*	Lines 235-236: this directly relates to the core of the author's claim: without the modulation through insulin-like peptides there is no modulation in the MB of the hot stimulus response. Hungry flies should have the same level of activation as satiated flies. Therefore, the two conditions (satiety and hunger), now plotted separately Figure 2 I-J, should be plotted together as in Figure 1 H, I, J.
Author’s response: Thank you for your comments. We have only compared the live brain calcium imaging experiments that were parallelly performed on the same day. Therefore, we cannot directly compare Fig 2I and Fig 2J since these are the results of two different experiments conducted on different days. For our revised manuscript, we parallelly performed the experiments to compare the hot responses in α'β' MBn in *InR^DN^* expressing flies (Fig 6H) or in *dilp2^-/-^* and *dilp6^LOF^* double mutant flies (Fig 6I). The results showed no significant difference in hot responses in α'β' MBn between sated and hungry flies when InR is inhibited in α'β' MBn (Fig 6H) or in *dilp2^-/-^* and *dilp6^LOF^* double mutants (Fig 6I).

*	Lines 278-279: Please be consistent with your statement in the introduction "levels of Dilp2 decrease in starved flies"
Author’s response: Thank you for your comment. We have modified the statement in the introduction as suggested. Lines 89–90: “Dilp2 secretion is dependent on the nutritional status; moreover, nutrient deprivation inhibits the secretion of Dilp2 by IPCs [16, 17]”.

*	Lines 281-282: the effects on HAB were present not only when alpha1beta1 was silenced but also when gamma and alphabeta were silenced in satiated flies (Figure 1 E F). Therefore, dilp2 phenotype in satiated flies should be checked in these lines, too.  
Author’s response: Our results showed that HAB is not affected in *VT44966-GAL4* (γ MBn) > *UAS-InR^DN^*, *R16A06-GAL4* (γ MBn) > *UAS-InR^DN^*, *VT49246-GAL4* (αβ MBn) > *UAS-InR^DN^,* and *C739-GAL4* (αβ MBn) > *UAS-InR^DN^* flies when compared to the HAB in their respective controls in the sated state (S4B-S4E Fig). The inhibition of the InR signal in γ or αβ MBn did not affect HAB, suggesting that Dilp modulations in αβ and γ MBn are not involved in regulating HAB.

*	Lines 285-286 and 295-296 are somewhat confusing.  Please clarify what mediates hot responses by bringing information about the internal state of the animal. A more comprehensive explanation of the reasoning would be beneficial to the reader. 
Author’s response: We have added the following statement to the results section for more clarity: “We performed live brain imaging to determine whether the Dilp2 secretion by IPCs represent satiety or a hot signal .” (Lines: 308­–309)

*	Lines 361-362: this is clearly not true as Figure 3 E shows that overexpression of Dilp2 results in a decrease of HAB, unless I have a misunderstanding…
Author’s response: We are grateful for your comments. We have revised the statement to: “The genetic silencing of *dilp2* in IPCs or inhibition of PI3K/AKT signaling in α'β' MBn increased HAB in food-sated but not in hungry flies (Figs 3C, 3D, 3G,3H, S6B, S6C and S7A-S7G)” (Lines: 373–375).

*	I suggest moving the Kir 2.1 manipulations to Supplementary to make panels easier to understand. 
Author’s response: We have moved the Kir 2.1 manipulations from the main figure to Figure S10E as suggested.

*	Finally, and although I try to not ask for new experiments, I would really like to see the 23 degrees and 25 degrees points in the plots as in Figure 1 B (perhaps the authors have those data already).

Author’s response: The distribution of flies on each plot in 23°C vs. 25°C and 25°C vs. 25°C experimental settings is shown below (Figure R1):

**Figure R1. Flies’ distributions on aluminum plate in 23°C vs 25°C and 25°C vs 25°C experimental settings.**

The two-choice assay plots (the top panel) of 23°C vs. 25°C (first row) and 25°C vs. 25°C (second row) in both feeding states. Data are represented as means ± SEM with dots representing individual values (P<0.0001 and 0.7543). Data were analyzed by the unpaired two-tailed *t*-test. **P*<0.05.

**References:**

Tsao CH, Chen CC, Lin CH, Yang HY, Lin SW. *Drosophila* mushroom bodies integrate hunger and satiety signals to control innate food-seeking behavior. Elife. 2018;7. doi:10.7554/eLife.35264.

Siju KP, Stih V, Aimon S, Gjorgjieva J, Portugues R, Grunwald Kadow IC. Valence and state-dependent population coding in dopaminergic neurons in the fly mushroom body. Curr Biol. 2020;30(11):2104-15 e4. doi: 10.1016/j.cub.2020.04.037.

Yamagata N, Hiroi M, Kondo S, Abe A, Tanimoto H. Suppression of dopamine neurons mediates reward. PLoS Biol. 2016;14(12):e1002586.doi: 10.1371/journal.pbio.1002586.

Shih HW, Wu CL, Chang SW, Liu TH, Sih-Yu Lai J, Fu TF, et al. Parallel circuits control temperature preference in *Drosophila* during ageing. Nat Commun. 2015;6:7775. doi: 10.1038/ncomms8775.
