# Supplementary material for: Independent insulin signaling modulators govern hot avoidance under different feeding states
Source: PLoS Biol. 2023 Oct 17;21(10):e3002332. doi: 10.1371/journal.pbio.3002332 (PMC10581474; doi:10.1371/journal.pbio.3002332)
Supplement: Attachment — Submitted filename: Response to Reviewers.docx [file pbio.3002332.s017.docx]

Please address the following editorial requests: 

1) The Academic Editor has one las suggestion which we think you should consider before publication. S/he says: "To future-proof your study, I suggest that you address the following editorial point, which does not require extra experiments but you may wish to consider it in the Discussion. Have you cross-checked the Ilp2 and Ilp6 manipulations with BOTH signalling pathways i.e not just p-Akt for Ilp2 and p-ERK for Ilp6? If not, it may be worth keeping open the possibility that Ilp2 may also increase p-ERK and Ilp6 also increase p-Akt?"

Author’s response:

We have added this following sentence in the Discussion part as suggested.” Our results further suggest that Dilp2 increases pAKT levels while Dilp6 increases pERK levels in α′β′ MBn during sated and hungry states (Figs 5E and 5F). However, we cannot totally rule out the possibility that Dilp2 also increases pERK levels and Dilp6 also increases pAKT levels in α′β′ MBn.” (Lines 614~618).

2) DATA REQUEST: Thank you for providing an S1_data file, which I assume has the underlying data related to your manuscript. As this was submitted as a .Rar file, I had trouble opening it. Can you please provide this as an excel file so I check that it meets our requirements?

Author’s response:

We have uploaded a new S1_data file with Excel format as suggested.

REVIEWS 
(note, we only sent the revision back to Reviewer 2, as the Academic Editor assessed the response to Reviewers 1 and 3)

Reviewer #2: The authors have addressed my comments in full and I have no further concerns. I wish to congratulate the authors for this interesting and rigorous study.

Author’s response:

Author’s response:

We are very grateful for your comments of our manuscript.
